# Postural Balance in Individuals With Knee Osteoarthritis During Stand-to-Sit Task

**DOI:** 10.3389/fnhum.2021.760960

**Published:** 2021-11-03

**Authors:** Shengxing Fu, Tingjin Duan, Meijin Hou, Fengjiao Yang, Yatai Chai, Yongkang Chen, Benke Liu, Ye Ma, Anmin Liu, Xiangbin Wang, Lidian Chen

**Affiliations:** ^1^National-Local Joint Engineering Research Center of Rehabilitation Medicine Technology, Fuzhou, China; ^2^Key Laboratory of Orthopedics and Traumatology of Traditional Chinese Medicine and Rehabilitation Ministry of Education, Fujian University of Traditional Chinese Medicine, Fuzhou, China; ^3^Department of Physical Education, Fujian University of Traditional Chinese Medicine, Fuzhou, China; ^4^College of Rehabilitation Medicine, Fujian University of Traditional Chinese Medicine, Fuzhou, China; ^5^Faculty of Sports Sciences, Research Academy of Grand Health, Ningbo University, Ningbo, China; ^6^School of Health and Society, University of Salford, Salford, United Kingdom

**Keywords:** knee osteoarthritis, stand-to-sit, postural balance, motion analysis, electromyography

## Abstract

**Objective:** Stand-to-sit task is an important daily function, but there is a lack of research evidence on whether knee osteoarthritis (knee OA) affects the postural balance during the task. This study aimed to compare individuals with knee OA and asymptomatic controls in postural balance and identify kinematic and lower extremity muscle activity characteristics in individuals with knee OA during the stand-to-sit task.

**Methods:** In total, 30 individuals with knee OA and 30 age-matched asymptomatic controls performed the 30-s Chair Stand Test (30sCST) at self-selected speeds. Motion analysis data and surface electromyography (sEMG) were collected while participants performed the 30sCST. To quantify postural balance, the displacement of the center of mass (CoM) and the peak instantaneous velocity of the CoM were calculated. The kinematic data included forward lean angles of the trunk and pelvic, range of motion (RoM) of the hip, knee, and ankle joints in the sagittal plane. The averaged activation levels of gluteus maximus, vastus lateralis, vastus medialis, rectus femoris, biceps femoris (BF), tibialis anterior (TA), and medial head of gastrocnemius muscles were indicated by the normalized root mean square amplitudes.

**Results:** Compared with the asymptomatic control group, the knee OA group prolonged the duration of the stand-to-sit task, demonstrated significantly larger CoM displacement and peak instantaneous CoM velocity in the anterior-posterior direction, reduced ankle dorsiflexion RoM, greater anterior pelvic tilt RoM, and lower quadriceps femoris and muscles activation level coupled with higher BF muscle activation level during the stand-to-sit task.

**Conclusion:** This study indicates that individuals with knee OA adopt greater pelvic forward lean RoM and higher BF muscle activation level during the stand-to-sit task. However, these individuals exist greater CoM excursion in the anterior-posterior direction and take more time to complete the task. This daily functional activity should be added to the rehabilitation goals for individuals with knee OA. The knee OA group performs reduced ankle dorsiflexion RoM, quadriceps femoris, and TA activation deficit. In the future, the rehabilitation programs targeting these impairments could be beneficial for restoring the functional transfer in individuals with knee OA.

## Introduction

Knee osteoarthritis (knee OA) is the most common degenerative joint disease, affecting an estimated 18% population in China (Wang et al., 2018). The disease is associated with pain, joint stiffness, quadriceps weakness, instability, and functional disability (Hunter and Bierma-Zeinstra, 2019).

In daily life, walking function is the basic activity, while sit-to-stand and stand-to-sit tasks can be the prerequisite and termination of gait, respectively. As people get older, sit and stand transition becomes a more demanding functional daily task (Galan-Mercant and Cuesta-Vargas, 2013). In fact, sit-to-stand or stand-to-sit motion variability has been proved to be significantly correlated with the risk of falling (Ghahramani et al., 2020). People suffering from knee OA have a higher prevalence of falls compared to non-OA subjects (Deng et al., 2021). Therefore, analyzing sit and stand transition and developing targeted rehabilitation plans can help individuals with knee OA perform the abovementioned tasks and reduce fall risk.

Sit-to-stand and stand-to-sit are sagittal planes dominant tasks. At present, the biomechanical characteristics of the sit-to-stand task such as kinetics, kinematics, and electromyography in the sagittal plane have been studied extensively in individuals with knee OA (Sonoo et al., 2019). The meta-analysis showed that individuals with knee OA tend to stand up with a lower knee extension moment during the sit-to-stand task (Sonoo et al., 2019). Moreover, individuals with knee OA demonstrated larger trunk flexion angle and forward center of mass (CoM) displacement (Naili et al., 2018; Sonoo et al., 2019). In neuromuscular activation level, individuals with knee OA activate more type II fibers of rectus femoris (RF) or increase the muscle activity of hamstrings (Bouchouras et al., 2015; Anan et al., 2016). These abovementioned biomechanical alterations indicate individuals with knee OA cannot perform the sit-to-stand task efficiently.

However, fewer studies have considered the stand-to-sit task. Unlike sit-to-stand, stand-to-sit is directly linked to the opposite movement and different muscle activation patterns (Ashford and De Souza, 2000). The stand-to-sit task requires almost simultaneous control of the anterior-posterior and vertical displacement of body CoM against gravity (Kerr et al., 1997). Trunk anteflexion and ankle dorsiflexion (Nakagawa and Petersen, 2018) play important roles during the stand-to-sit task to control the CoM backward and downward. Previous research reported women with knee OA showed smaller ankle dorsiflexion angles during the stand-to-sit task (Wu et al., 2015). Moreover, the eccentric contraction of the knee and hip extensors is essential in slowing down the movement velocity to enable a stable and safe landing to the seat (Ferrante et al., 2005). Another study that found women with knee OA exists weaker vastus lateralis (VL) activation combined with reduced knee flexion range of motion (RoM) during the stand-to-sit task (Bouchouras et al., 2020). These abovementioned motor changes and muscle activation alternations may bring challenges to individuals with knee OA with the increased risk to fall back to the seat.

Proper balance is essential to perform the stand-to-sit task and prevent high impact forces during seat contact that would lead to increased impact to the spine (Chen et al., 2010; Sibley et al., 2013). Thus, the importance of achieving movement control during the stand-to-sit task should not be underestimated for individuals with knee OA. In addition, there is still a lack of evidence to explore trunk motion and hip or ankle muscle activity in individuals with knee OA during the stand-to-sit task. Illustrating the postural balance and kinematic and muscle activity characteristics during the stand-to-sit task in individuals with knee OA is essential for customizing rehabilitation goals and restoring functional transfer.

The 30-s Chair Stand Test (30sCST) is one of the five physical function tests recommended for people with knee OA by The Osteoarthritis Research Society International (OARSI; Dobson et al., 2013). Compared to the five-repetition stand-to-sit test, performing as many stand-to-sit repetitions as possible during 30sCST is easier to fully capture the impaired postural balance and biomechanical alterations.

Therefore, the purpose of this study was to investigate the impact of knee OA on postural balance and identify kinematic and lower extremity muscle activity characteristics in individuals with knee OA during the descending phase of 30sCST. The primary outcome of this study was CoM displacement and velocity. The secondary outcomes included the duration of the stand-to-sit task, segment RoM, and lower extremity muscle activation level. We hypothesized that the individual with knee OA would display larger CoM displacement or velocity, longer task duration together with different movement strategies, and lower extremity muscle activity alternations during the task (Wu et al., 2015; Bouchouras et al., 2020).

## Materials and Methods

### Participants

Individuals with unilateral or bilateral mild-moderate (II or III Kellgren/Lawrence (K/L) grade) knee OA were the focus group in this study. To identify the performance variation, a control group with age-matched asymptomatic individuals was included in this study. By referring to similar research (Bouchouras et al., 2020), we used the power of 0.8, the effect size (ES) of 0.75, and two-sided α = 0.05 to calculate the sample size. G × Power software (version 3.1.9.2, Franz Faul, University of Kiel) showed that a minimum number of 29 participants per group should be obtained. Finally, 30 participants for each group were recruited from the neighboring communities of the Fujian University of Traditional Chinese Medicine (FJTCM) *via* advertisements in print/radio/social media.

The following inclusion criteria were set for the individuals with knee OA: fulfilled with the clinical diagnosis of 2018 Diagnosis and Treatment of Osteoarthritis (Osteoporosis Group of Chinese Orthopedic Association, 2018) and had II or III K/L grade. The K/L grade of individuals with knee OA was defined by anterior X-ray images of identified osteophytes and narrowing of the joint space in a standing position (Ribeiro et al., 2020). The inclusion criteria of the control group were age-matched people without knee OA-related symptoms and any other conditions that would affect walking and postural balance. Participants of both groups were able to accomplish sit and stand transitions without assistive devices. Participants were excluded if they had other lower extremity joint pain, severe back pain, rheumatoid arthritis, fractures, neurological system pathology, or obesity (body mass index (BMI) > 28 kg/m^2^) (Li et al., 2020).

The experiment protocol was approved by the Ethics Committee of the Affiliated Rehabilitation Hospital of FJTCM (#2018KY-006-1) and registered on the Chinese Clinical Trial Registry website (identifier number ChiCTR1800018028)^1^. All participants were informed about the study protocol as well as potential benefits and risks and provided written and oral consent prior to the experiment.

### Data Collection

Lower extremity muscle activities were measured with a wireless surface electromyography (sEMG) system (Trigno Wireless EMG System, Delsys Inc., Natick, MA, United States) at a sampling frequency of 2,000 Hz and a band-pass filter of 20–450 Hz. Skin preparation and location of the electrodes followed the recommendations of sEMG for the Non-Invasive Assessment of Muscles (SENIAM) (Hermens et al., 2000). NuPrep skin preparation gel is beneficial for use where motion artifacts can affect readings, and when a reduction of skin impedance would enhance a test result. Adhesive pre-gelled Ag/AgCl electrodes (Trigno Avanti Sensor, Delsys, United States) were placed bilaterally on the gluteus maximus (GMAX), VL, vastus medialis (VM), RF, biceps femoris (BF), tibialis anterior (TA), and medial head of gastrocnemius (MG) muscles.

**FIGURE 1:**
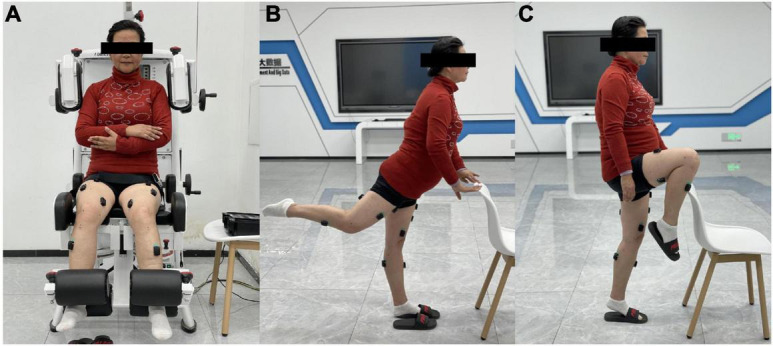
Surface electromyography (sEMG) amplitude normalization tests: **(A)** for vastus lateralis/vastus medialis/rectus femoris/biceps femoris/tibialis anterior (VL/VM/RF/BF/TA), **(B)** for gluteus maximus (GMAX), and **(C)** for medial head of gastrocnemius (MG).

After the placement of electrodes, three amplitude normalization tests were performed for each investigated muscle separately to direct quantitative comparison of sEMG data between participants. Before the normalization tests, each participant performed the initial warming up sequence (stretching, 5 min). During each muscle normalization test, participants followed visual (looking at the real-time sEMG curve on the screen) and verbal stimulation, slowly started increasing the force, reached the maximum effort, and held it for 3 s, and promptly relaxed (Anan et al., 2016). Each muscle repeated the normalization test three times with a pausing period of 30–60 s in between. For VL/VM/RF/BF/TA, the normalization tests adopted the gold standard that is the maximum voluntary isometric contraction (MVC) test. The MVC tests were measured at the muscle strength test system (Myonline Professional, DIERS International GmbH, Germany). The starting position was standardized with the participants seated on the device with the pelvis as close as possible to the backrest. The lower legs were set between the two leg extension/flexion pads, and the participant was secured firmly using the pelvic/hip strap and the thigh strap. Then, each participant performed maximal knee extension, knee flexion, and ankle dorsiflexion against the rear pad around the ankle joint successively (Figure 1A). When one leg was measured, the other leg was supported in a relaxed position. Due to the fact that the Myonline equipment could not complete the GMAX and MG MVC tests, the GMAX and MG normalization tests were replaced by the isometric contraction against gravity in a standing position. The alternative normalization test also has good reliability (Burden, 2010). For GMAX, the participants slowly extended one hip joint to the highest height as possible with the upper body upright (Figure 1B). For MG, the participants performed as follows: one lower limb was tiptoe while the other side was off the ground (Figure 1C).

**FIGURE 2:**
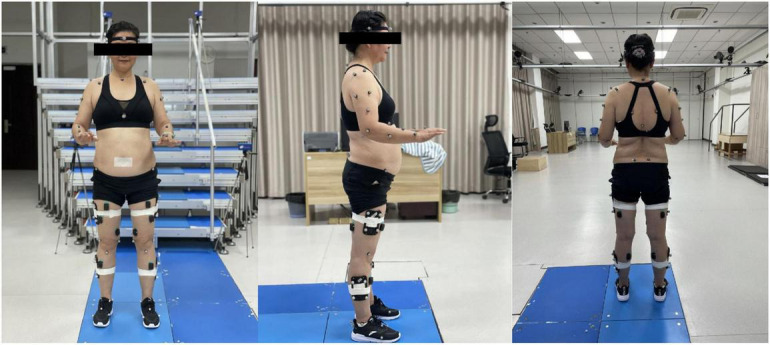
The placement of the markers.

After the normalization tests, 75 retroreflective markers were placed on the anatomical landmarks and top of each segment as tracking markers (Table 1 and Figure 2). The placement of the marker was according to the calibrated anatomical systems technique protocol (Cappozzo et al., 1995) to form a 15-segment whole-body model. The kinematic data were collected by a 3D motion capture system equipped with a 10-camera setup (Oqus 7+, Qualisys AB, Sweden) at a sampling rate of 100 Hz. After the placement of the reflective markers, a static standing trial was recorded to create a model of the participant in Visual 3D.

**TABLE 1:** Seventy-five retroreflective markers placement.

**Marker name**	**Marker location**
**Upper body**	
L/R_HEAD	Just above the ear
SGL	Glabella
CLAV	Clavicular notch
STRN	Sternum
CV7	7th Cervical Vertebrae
TV10	10th Thoracic Vertebrae
L/R_SIA	Scapula-Inferior Angle
L/R_SAE	Scapula-Acromial Edge
L/R_ASH	Anterior shoulder
L/R_PSH	Posterior shoulder
L/R_1-3 Cluster	Cluster of three markers placed on the lateral surface of the upper arm
L/R_HLE	Humerus – Lateral Epicondyle
L/R_HME	Humerus – Medial Epicondyle
L/R_1-3 Cluster	Cluster of three markers placed on the lateral surface of the forearm
L/R_RSP	Radius – Styloid Process
L/R_USP	Ulna – Styloid Process
L/R_HM2	Basis of Forefinger
**Lower body**	
L/R_IAS	Anterior superior iliac spine
L/R_IPS	Posterior superior iliac spine
L/R_TH1-4 Cluster	Cluster of four markers placed on the lateral surface of the thigh
L/R_FLE	Lateral epicondyle
L/R_FME	Medial epicondyle
L/R_TT	Tuberositas tibiae
L/R_SK1-4 Cluster	Cluster of four markers placed on the lateral surface of the shank
L/R_FAL	Lateral prominence of the lateral malleolus
L/R_TAM	Medial prominence of the medial malleolus
L/R_FCC	Aspect of the Achilles tendon insertion on the calcaneus
L/R_FM1	Dorsal margin of the first metatarsal head
L/R_FM2	Dorsal aspect of the second metatarsal head
L/R_FM5	Dorsal margin of the fifth metatarsal head

**FIGURE 3:**
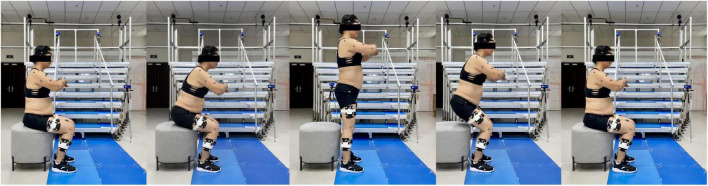
The 30-s Chair Stand Test (30sCST).

Participants were instructed to perform 30sCST while wearing sports shoes (112027711-4/122025523R-2, Anta Co. Ltd., China) (Figure 3). Each participant performed the 30sCST under the following instructions: (1) started from the seated position, the feet were allowed to be placed flat on the floor and shoulder-width apart, arms crossed on chest, stood completely up, and then sat completely back down and (2) rising at the natural speed of the participant and as fast as possible during 30 s. An armless chair with a standardized seat height of approximately 43 cm (17-inch) was used according to the OARSI (Dobson et al., 2013). The seat was placed on an anti-slip surface. This process was performed with each leg on one force plate. Ground reaction forces (GRFs) were simultaneously measured using force plates with a sampling rate of 2,000 Hz (9260AA, Kistler Ltd., Switzerland). Some practice trials were performed prior to the test by all participants to familiarize themselves with the 30sCST.

### Data Analysis

All data processing and outcome calculations were in Visual3D (V6, C-motion Inc., Germantown, MD, United States). The marker data were filtered using a fourth-order low-pass Butterworth filter with a cutoff frequency of 6 Hz (Robertson and Dowling, 2003). The GRF raw data were filtered with a fourth-order low-pass Butterworth filter with a cutoff frequency of 20 Hz (Piano et al., 2020). Both primary and secondary outcomes, i.e., the CoM displacement, peak velocity and the stand-to-sit task time, segment RoM, and lower extremity muscle activation level, were calculated based on the filtered data.

Segment coordination systems of the trunk, pelvis, both thighs, shanks, and feet were defined based on the anatomical markers (Robertson et al., 2013). Hip, knee, and ankle joint angles were defined as the angle between proximal and distal segments. Trunk segment angle and pelvic segment angle were determined with respect to the laboratory coordinate system (Jeon et al., 2021). Joint angles were calculated with a Cardan x–y–z (mediolateral, anteroposterior, and transverse) rotation sequence (Cole et al., 1993). The forward lean RoM of the trunk and pelvic and the RoM of the hip, knee, and ankle joints in the sagittal plane were calculated using Visual3D.

The CoM was calculated using the weighted average of all the segments of the body according to the study by Robertson et al. (2013). The peak-to-peak displacement of CoM and the peak instantaneous velocity of the CoM in anterior-posterior and vertical directions were used to quantify the body oscillation during dynamic functional tasks (Hsue and Su, 2014). An increased value for either variable suggests a decreased ability to maintain balance (Hsue and Su, 2014).

Motion initiation was defined as the first transition from negative to positive trunk angular velocity after the occurrence of the maximum knee extension angle (Anan et al., 2015). Motion termination was defined as the instant vertical vector of the GRF less than 10 N (Anan et al., 2015). The duration of the stand-to-sit task was the time interval between the movement initiation and termination.

The sEMG signals of the normalization tests and the stand-to-sit task were full-wave rectified and enveloped with a root mean square (RMS) algorithm with a 50-ms window (Anan et al., 2016). Each sEMG signal during the stand-to-sit task was normalized to the corresponding peak value of three normalization tests. The RMS value of each normalized sEMG signal was calculated during the stand-to-sit task to quantify the magnitude of the muscle excitation.

Each index was represented using the mean value of many stand-to-sit transitions during the stable period (10–25 s) of 30sCST. For lower extremity muscle activity and joint RoM, we compared the more affected leg of the knee OA group and the dominant side leg of the control group. The dominant side leg is defined as the preferred limb when kicking a ball (van Melick et al., 2017). If bilateral symptomatic individuals with knee OA have similar knee pain on both sides, we would choose the dominant limb.

### Statistics

All values are presented as mean ± SD. Prior to all analyses, the normality of the quantitative data was assessed using the Shapiro–Wilk test. The two independent samples *t*-test was used to compare continuous normally distributed variables, i.e., age, height, body mass, BMI, CoM parameters, segment RoM, and BF muscle activation level. The Wilcoxon Mann–Whitney *U* test was used to compare the non-normal variables, i.e., stand-to-sit time and muscle activation level except for BF. A chi-square test was used to compare the qualitative data, i.e., gender. IBM SPSS version 25.0 (SPSS Inc., Chicago, IL, United States) was used for all statistical analyses. The significance level was set at less than 0.05. To determine the magnitude of difference between the two groups, ES calculations (Cohen’s *d* for quantitative data and Cramer’s φ for qualitative data) were reported for all measures. An ES from 0.1 to 0.3 was regarded as a small effect, 0.3–0.5 as intermediate, >0.5 as a strong effect (Cohen, 2013).

## Results

### Participants

In total, 60 participants completed the study (knee OA group: *n* = 30; control group: *n* = 30). There was no significant difference between the groups for age (knee OA group: 58.63 ± 5.67 vs. control group: 59.33 ± 5.14 years, *P* = 0.618, ES = 0.129), height (1.60 ± 0.56 vs. 1.60 ± 0.67 m, *P* = 0.900, ES = 0), body mass (59.13 ± 7.56 vs. 59.00 ± 9.63 kg, *P* = 0.952, ES = 0.015), BMI (23.08 ± 2.54 vs. 22.85 ± 2.41 kg/m^2^, *P* = 0.721, ES = 0.096), and the male/female ratio (4/26 vs. 8/22, *P* = 0.197, ES = 0.167; Table 2).

**TABLE 2:** Characteristics of the knee osteoarthritis (OA) and control groups.

	**knee OA group (*n* = 30)**	**Control group (*n* = 30)**	***P*-value**	**Effect size**
Age	58.63 ± 5.67	59.33 ± 5.14	0.618	0.129
Height (m)	1.60 ± 0.56	1.60 ± 0.67	0.900	0
Body mass (kg)	59.13 ± 7.56	59.00 ± 9.63	0.952	0.015
BMI (kg/m^2^)	23.08 ± 2.54	22.85 ± 2.41	0.721	0.096
Gender (male/female)	4/26	8/22	0.197	0.167
K/L grade (II/III)	23/7	/	/	/
Course of disease (month)	93.10 ± 86.74	/	/	/

*Mean ± SD.*

*BMI, body mass index; K/L, Kellgren/Lawrence.*

### Stand-to-Sit Task Time

The stand-to-sit task time in the knee OA group and control group was 0.95 ± 0.15 and 0.81 ± 0.20 s, respectively. The knee OA group showed a statistically significant longer task time (*P* < 0.001, ES = 0.849; Table 3).

**TABLE 3:** Data between the knee OA and control groups.

	**knee OA group (*n* = 30)**	**Control group (*n* = 30)**	***P*-value**	**Effect size**
Stand-to-sit time (s)	0.96 ± 0.15	0.81 ± 0.20	**<0.001**	**0.849**
**CoM parameters**				
d_CoM, AP_ (m)	0.19 ± 0.04	0.16 ± 0.04	**0.002**	**0.750**
d_CoM, vertical_ (m)	0.24 ± 0.03	0.24 ± 0.03	0.800	0
V_CoM, AP_ (m/s)	0.38 ± 0.07	0.34 ± 0.07	**0.029**	**0.571**
V_CoM, vertical_ (m/s)	0.54 ± 0.10	0.58 ± 0.11	0.172	−0.381
**RoM (°) in sagittal plane**				
Trunk	28.49 ± 7.64	25.05 ± 5.72	0.053	0.510
Pelvic	21.99 ± 4.75	18.32 ± 5.11	**0.005**	**0.744**
Hip	74.56 ± 11.54	69.67 ± 7.35	0.056	0.505
Knee	80.57 ± 10.12	80.29 ± 8.86	0.910	0.029
Ankle	11.80 ± 5.35	15.10 ± 4.75	**0.014**	−**0.652**
**RMS (%) of muscle**				
GMAX	4.03 ± 2.64	2.89 ± 1.34	0.065	0.545
VL	13.11 ± 5.13	17.78 ± 8.09	**0.032**	−**0.689**
VM	14.96 ± 5.68	20.04 ± 9.57	**0.035**	−**0.646**
RF	8.93 ± 4.69	12.87 ± 7.15	**0.022**	−**0.652**
BF	5.01 ± 2.50	3.81 ± 1.69	**0.034**	**0.562**
TA	7.59 ± 4.67	10.18 ± 4.71	**0.017**	−**0.552**
MG	3.56 ± 2.25	3.29 ± 2.87	0.329	0.105

*Mean ± SD.*

*CoM, center of mas; d_*CoM, AP*_, displacement of CoM in anterior-posterior direction; d_*CoM, vertical*_, displacement of CoM in vertical direction; V_*CoM, AP*_, velocity of CoM in anterior and posterior direction; V_*CoM, vertical*_, velocity of CoM in vertical direction; RMS, root mean square; GMAX, gluteus maximus; VL, vastus lateralis; VM, vastus medialis; RF, rectus femoris; BF, biceps femoris; TA, tibialis anterior; MG, medial head of gastrocnemius. The bolded values mean the *P*-value is smaller than 0.05.*

### Center of Mass Parameters

In the anterior-posterior direction, the results from the study demonstrated that the CoM displacement of the knee OA group was 0.03 m larger than that of the control group (0.19 ± 0.04 vs. 0.16 ± 0.04 m, *P* = 0.002, ES = 0.750) during the stand-to-sit task. Also, the peak instantaneous velocity of CoM in the anterior-posterior direction of the knee OA group was 0.04 m/s higher than that of the control group (0.38 ± 0.07 vs. 0.34 ± 0.07 m/s, *P* = 0.029, ES = 0.571). Whereas in the vertical direction, there was no statistically significant difference of CoM displacement and peak instantaneous velocity between the two groups (*P* > 0.05; Table 3).

### Segment Range of Motion

The pelvic forward lean RoM in the knee OA group was significantly larger than that in the control group (21.99° ± 4.75° vs. 18.32° ± 5.1°, *P* = 0.005, ES = 0.744). In addition, the knee OA group presented smaller ankle dorsiflexion RoM (11.80° ± 5.35° vs. 15.10° ± 4.75°, *P* = 0.014, ES = −0.652). The trunk forward lean RoM and hip and knee flexion RoM showed no statistically significant difference between the two groups (*P* > 0.05; Table 3).

### Muscle Activation Level

There were smaller RMS values of VL, VM, RF, and TA muscles in the knee OA group compared to the control group (VL: 13.11 ± 5.13 vs. 17.78 ± 8.09%, *P* = 0.032, ES = −0.689; VM: 14.96 ± 5.68 vs. 20.04 ± 9.57%, *P* = 0.035, ES = −0.646; RF: 8.93 ± 4.69 vs. 12.87 ± 7.15%, *P* = 0.022, ES = −0.652; TA: 7.59 ± 4.67 vs. 10.18 ± 4.71%, *P* = 0.017, ES = −0.552). Meanwhile, BF muscle in the knee OA group showed larger RMS value than that in the control group (5.01 ± 2.50 vs. 3.81 ± 1.69%, *P* = 0.034, ES = 0.562). There was no statistically significant difference in the RMS values of GMAX and MG between the two groups (*P* > 0.05; Table 3).

## Discussion

This study aimed to investigate the influence of knee OA on postural balance and investigate the differences in the measures of the trunk, pelvic, lower extremity kinematics, and lower extremity muscle activity between the knee OA group and the control group during the stand-to-sit task. We found that individuals with knee OA showed greater postural sway and prolonged duration of the stand-to-sit task, reduced ankle dorsiflexion RoM, quadriceps femoris, and TA activation level during the stand-to-sit task in comparison with the control group. At the same time, individuals with knee OA may increase pelvic anterior tilt RoM and BF muscle activity to functional compensation than the control group during the task.

The meta-analysis showed that individuals with knee OA had significantly longer sit-to-stand times (Sonoo et al., 2019). Longer task time is associated with limited physical function (Segal et al., 2013). However, few studies reported the duration of the stand-to-sit task in individuals with knee OA. It was previously reported that there was no statistically significant difference in task duration between women with knee OA and healthy subjects during three sittings (Bouchouras et al., 2020). In our study, results showed that individuals with knee OA took more time to accomplish the stand-to-sit task. The ability to perform the stand-to-sit task is influenced by knee OA disease. 30sCST seemed to be challenging enough to capture the impaired function in individuals with knee OA compared with the three-repetition stand-to-sit task.

With regard to postural stability, the results demonstrated that the knee OA group had greater CoM displacement and peak instantaneous velocity in the anterior-posterior direction, which could be an indication that individuals with knee OA would have a greater risk to fall backward. Some researchers have reported that individuals with knee OA showed impaired balance in other daily activities such as standing (Truszczyńska-Baszak et al., 2020), walking (Graber et al., 2021), and stair descending (Koyama et al., 2015). Poor balance is related to muscle weakness in individuals with knee OA (Bennell et al., 2011). The stand-to-sit task is performed with an eccentric contraction of the knee and hip extensors to slow down the movement velocity (Ferrante et al., 2005). However, due to disuse atrophy and reflex inhibition caused possibly by pain, knee OA would result in deficits in the voluntary activation of the quadriceps femoris (Kittelson et al., 2014). Our results demonstrated VL, VM, and RF activation deficit in individuals with knee OA during the stand-to-sit task, which conformed to the findings from previous research (Bouchouras et al., 2020). Quadriceps eccentric contraction exercise may need to be addressed for individuals with knee OA to improve physical balance.

Moreover, the results from this study showed that the knee OA group demonstrated reduced ankle dorsiflexion RoM and lower muscle activity of TA. The result of reduced ankle dorsiflexion RoM in our study was similar to the result of a previous study (knee OA: 13.2° ± 6.3° vs. control: 15.8° ± 5.2°) (Wu et al., 2015). The dynamic balance could be influenced by the change in the ankle movement during weight-bearing activities. It was reported that the reduced ankle dorsiflexion RoM was correlated with instability along the anterior-posterior direction and would affect the ability to lower the CoM of the body (Nakagawa and Petersen, 2018). In addition, limitations in ankle dorsiflexion showed that it could result in knee abnormal alignment and increase the risk for knee joint pathology (Basnett et al., 2013; Lima et al., 2018). TA muscle is the active muscle that produces ankle dorsiflexion, which had been rarely studied in individuals with knee OA before this study. A previous study concluded that knee OA would lead to a decrease in TA muscle contractile tissue (Taniguchi et al., 2015), which may influence TA muscle activation. Our study demonstrated TA muscle activation deficit in individuals with knee OA. Future studies should be performed to determine whether interventions directed at improving ankle dorsiflexion RoM and TA muscle activation would have an effect on postural balance.

The human body generally takes compensation strategies to maintain equilibrium when postural sway happens during daily activity. Trunk and pelvic anteflexion would control the backward movement of the CoM (Takeda, 2012; Darwish et al., 2019). During the sit-to-stand task, individuals with knee OA were found to have an increased trunk flexion angle to move CoM forward (Sonoo et al., 2019). In our study, we found that individuals with knee OA adopted another strategy, which was reflected as a greater pelvic anterior tilt angle during the stand-to-sit task. Greater pelvic anterior tilt could keep the CoM within the base of support, and this enables the CoM to retain longer in the support area throughout the task to reduce the risk of falling back to the seat (Darwish et al., 2019). In contrast, this strategy characterizes a method to reduce the quadriceps demand (Goncalves et al., 2017).

Increasing the BF muscle activity is a common appearance in individuals with knee OA during daily activities (Mills et al., 2013). The BF muscle activation provided the additional force to balance and stabilize the knee joint (Mills et al., 2013). Higher BF muscle activation level in our study could be the strategy to compensate quadriceps activation deficit, but this strategy would result in higher energetic costs or joint load (Hortobágyi and DeVita, 2000; Patsika et al., 2011). BF muscle is a two-joint muscle, originated from ischial tuberosity to the lateral aspect of the fibular head. From the perspective of the BF muscle anatomy, the other alternative explanation is that the greater pelvic anterior tilt results in the lengthening of the BF that leads to higher muscle activity.

### Limitation

First, the height of the chair was not adjusted to the lower leg length of the participants. We used a chair with a standardized seat height of approximately 17-inch according to the OARSI that has the tremendous advantage to reflect the real-life situation for elderly people. However, a previous study reported that chair seat height in relation to the lower leg length should be considered when interpreting 30sCST performance (Kuo, 2013). Second, we ignored unilateral/bilateral symptoms and the movements that may occur in the frontal plane, which may further explain impairments in postural balance. Moreover, there was unequal men/women representation, and the results of our study may not be applicable to men. Whether there are different performances between men and women remain to be studied. Finally, this study was a cross-sectional design, it may be hard to conclude the cause-and-effect relationships. Further research should evaluate the influence of rehabilitation on improving ankle dorsiflexion kinematic and lower extremity muscle activity in relation to postural balance.

## Conclusion

In our study, individuals with knee OA adopt greater pelvic forward lean RoM and higher BF muscle activation levels during the stand-to-sit task. However, these individuals still demonstrated greater CoM excursion in the anterior-posterior direction and took more time to complete the task. Knee OA leads to postural instability and functional disability during the stand-to-sit task. This daily functional activity should be added to the rehabilitation goals for individuals with knee OA. The healthcare professional should recommend that individuals with knee OA use an armrest or handrail to reduce the risk of falls during the stand-to-sit task. Our findings demonstrated that individuals with knee OA performed reduced ankle dorsiflexion RoM, quadriceps femoris, and TA activation deficit. The rehabilitation programs targeting these impairments could be beneficial for restoring the functional transfer in individuals with knee OA.

## Data Availability Statement

The raw data supporting the conclusion of this article will be made available by the authors, without undue reservation.

## Ethics Statement

The studies involving human participants were reviewed and approved by the Ethics Committee of the Affiliated Rehabilitation Hospital of Fujian University of Traditional Chinese Medicine, Fuzhou, Fujian, China (#2018KY-006-1). The patients/participants provided their written informed consent to participate in this study. Written informed consent was obtained from the individual(s) for the publication of any potentially identifiable images or data included in this article.

## Author Contributions

XW and LC: contributed to conceive, design, and obtain funding for the study. SF, TD, and MH: contributed to experimental design, data collection and analysis, and manuscript writing. FY, YaC, YoC, and BL: contributed to data collection and analysis. AL: contributed to the establishment of research questions, the discussion focus, and manuscript revision. YM: contributed to manuscript revision. All the authors were involved in the revision and final approval of the manuscript.

## Conflict of Interest

The authors declare that the research was conducted in the absence of any commercial or financial relationships that could be construed as a potential conflict of interest.

## Publisher’s Note

All claims expressed in this article are solely those of the authors and do not necessarily represent those of their affiliated organizations, or those of the publisher, the editors and the reviewers. Any product that may be evaluated in this article, or claim that may be made by its manufacturer, is not guaranteed or endorsed by the publisher.

## Footnotes

1
http://www.chictr.org.cn
